# Post-pancreatitis diabetes mellitus: insight on optimal management with nutrition and lifestyle approaches

**DOI:** 10.1080/07853890.2022.2090601

**Published:** 2022-07-04

**Authors:** Amandeep Singh, Manik Aggarwal, Rajat Garg, Tyler Stevens, Prabhleen Chahal

**Affiliations:** Department of Gastroenterology, Hepatology and Nutrition, Digestive Diseases and Surgery Institute, Cleveland Clinic, Cleveland, OH, USA

**Keywords:** Post-pancreatitis diabetes, diet, nutrition, management

## Abstract

Pancreatitis is the leading gastrointestinal cause of hospitalizations. There are multiple short- and long-term complications associated with pancreatitis. Post-pancreatitis diabetes mellitus (PPDM) is one of the less explored complications of pancreatitis. Nonetheless, it has attracted considerable attention during the last decade. PPDM is now the second most common cause of new-onset diabetes mellitus (DM) in adults after type II DM surpassing type 1 DM. However, there exists a knowledge gap amongst practitioners regarding diagnosis, complications, and management of PPDM. In this narrative, we aim to provide a brief review regarding risks, diagnosis and management of PPDM with a special focus on dietary and lifestyle management strategies.

**KEY MESSAGES**
1. Post-pancreatitis diabetes mellitus (PPDM) is now the second most common cause of new-onset diabetes mellitus (DM) in adults after type II DM surpassing type 1 DM.
2. New-onset diabetes in patients with pancreatitis could also be an early marker of occult pancreatic malignancy.
3. Management of PPDM is complex and requires a team-based approach including gastroenterologists, endocrinologists, primary care physicians, nutritionists, and behavioural health specialists.

## Introduction

Pancreatitis is one of the leading gastrointestinal aetiologies for hospitalization and is responsible for significant morbidity [1]. Worldwide, the rates of acute and chronic pancreatitis are 33.7 cases/100,000 person-year and 9.6 cases/100,000 person-year, respectively [2]. Severe acute pancreatitis can have mortality up to 30.0% [1]. Pancreatitis admissions account for over $2.5 Billion of health care spending per year [2]. The complications of acute pancreatitis (AP) including abdominal fluid collections, necrosis, and sepsis requiring interventions can prolong the length of hospital stay and add to the cost of hospitalization [2]. Severe cases may result in long-term complications including chronic pain, inability to tolerate oral intake, endocrine, and exocrine dysfunction. In recent years, the subject of post-pancreatitis diabetes mellitus (PPDM) also known as type-3c diabetes has garnered much attention from researchers. PPDM represents a long-term sequela of pancreatitis and is the most common disease of the exocrine pancreas [3]. An episode of pancreatitis confers a 2-fold elevation in risk of subsequent diabetes [4]. Furthermore, in patients with a history of pancreatitis, new-onset diabetes could also indicate undiagnosed pancreatic cancer [5]. Interestingly, PPDM confers a significantly higher risk of pancreatic cancer than pancreatitis in patients with pre-existing type 2 diabetes mellitus (T2DM) (hazard ratio [HR] 2.35; 95% confidence interval [CI] 1.12–4.93) [6,7].

PPDM is now the second most common cause of new-onset diabetes in adults after type T2DM surpassing type 1 DM (T1DM) [4]. In contrast with type 1 and type 2 DM, the epidemiology of PPDM has been relatively unknown until the last decade. The prevalence is still likely to be under-estimated and may be expected to rise with increasing awareness. Diabetes secondary to diseases of the exocrine pancreas (DEP) or ‘pancreatogenic’ diabetes constitutes 1.6%–1.8% of new-onset diabetes in adults in comparison with 1.1% for type1DM (T1DM) [8]. An overall incidence of 2.6–2.8 per 100,000 general population for PPDM has been reported in population-based studies from New Zealand and the United Kingdom [4].

Both acute and chronic pancreatitis (CP) carry the risk of the development of PPDM. Compared to T2DM, PPDM is associated with poor glycemic control, lower body mass index (BMI) at diagnosis, higher risk of cancer development, death at a younger age, and a significantly higher risk of mortality [3,6,9]. One of the possible reasons for higher complications and mortality rates associated with PPDM is under or misdiagnosis in the early stages leading to delayed diagnosis with unwanted complications. Clinico-pathological features and management challenges in PPDM overlap with other secondary causes of diabetes including cystic fibrosis related diabetes (CFRD). The complex pathogenesis involving exocrine pancreatic insufficiency (EPI), impaired insulin secretion and insulin resistance are shared between PPDM and CFRD [10].

There is a significant lack of knowledge amongst practicing providers about diagnosis, complications and management of PPDM. Prompt and correct diagnosis of this entity has far-reaching implications including counselling the patients, generating better management strategies to improve glycemic control to reduce short and long-term complications, increasing patient and provider satisfaction, and decreasing burnout amongst treating physicians [11]. In this review, we aim to provide an overview of risk factors, diagnostic criteria and management strategies for PPDM with a special focus on dietary and lifestyle management.

## Risks of PPDM

There are multiple underlying pathways leading to PPDM including insulin deficiency, inflammation/cytokine medicated β-cell dysfunction before β-cell loss, peripheral and hepatic insulin resistance mediated by pancreatic polypeptide, and reduced incretin response [12–14]. An aetiology that leads to functional or structural loss of glucose normalizing insulin secretion can lead to PPDM [15]. Risks for PPDM are divided into two major categories:

### Demographical risks

In terms of age, compared to the general population, individuals under the age of 40 with a history of AP have a higher risk of developing PPDM (adjusted odds ratio [aOR]: 4.65; 95%CI: 2.48–8.72) [16]. This is different from T2DM where middle-aged and older adults have the highest risk of developing T2DM [3]. Similarly, in contrast to T2DM, where men and women have an equivalent risk of developing T2DM, men are at a considerably higher risk of developing PPDM than women [4,17]. In a population-based study the risk of developing PPDM after an attack of AP was noted to be 2-fold higher than the general population and men had a significantly higher risk of developing PPDM than women (HR 3.21 vs. 1.58, *p* = .0004) [17]. In terms of body composition, the risk for PPDM increases in lean or overweight individuals. This again is different from T2DM where obesity or overweight is a key risk factor. A study from the United Kingdom demonstrated that the proportion of obese individuals was significantly lower (OR: 0.77; 95% CI:0.63–0.95) and the proportion of lean individuals was significantly higher (OR: 1.59; 95% CI:1.14–2.22) in PPDM cohort than patients with T2DM [4]. Increased visceral and intra-pancreatic fat volume have been seen in AP patients with DM in comparison with those without DM [18,19]. The role of intra-pancreatic fat in the development of PPDM remains to be elucidated and may serve as a diagnostic and/or therapeutic target in the future. A study by Goodarzi and colleagues compared genetic risk scores (GRS) based on validated single nucleotide polymorphisms between 321 subjects with CP-DM and 423 subjects with T2DM. There were no differences in GRS between the two groups, suggesting a shared risk profile between the two disease entities [20].

### Pancreas specific risk factors

#### Exocrine pancreatic insufficiency (EPI)

EPI, pancreatic ductal damage and stricture, and pancreatic calcifications are independent risk factors for PPDM. A recent nationwide study from New Zealand reported EPI posed a 3.8-times higher risk for the development of PPDM with a mean duration of 1.8-years between EPI and PPDM [21]. Vitamin D deficiency, altered gut-microbiome, and chronic inflammation resulting in insulin resistance play a pathogenic role in the development of PPDM. In addition, in contrast to T2DM where men and women have a similar risk of developing diabetes, men with EPI have a significantly higher risk of developing PPDM. The prevalence of PPDM is significantly higher for men than women in both acute and chronic pancreatitis (93.3% vs. 62.1% and 14.2% vs. 6.3% per 1,000 patients with EPI, respectively, *p*<0.05) [22].

#### Aetiology and severity of pancreatitis

Neither the severity of the initial episode nor the aetiology appears to be related to the risk of PPDM. A meta-analysis of 24 prospective studies showed the risk of PPDM was similar between patients with mild and severe AP. Similar results were reported in subsequent population-based studies with an average 2-fold higher risk of PPDM in both mild and severe AP [17,23]. Whether there is a transient “stunning” of islet cells in mild AP causing PPDM still needs to be explored. Prospective studies with long-term follow-ups are required to make a distinction between transient vs. permanent loss of islet cell function from pancreatitis episodes with various degrees of severities.

Although alcohol use is an established risk factor for pancreatitis, the evidence linking it to DM is mixed. Alcohol-related pancreatitis was earlier thought to portend higher risk for PPDM with a study demonstrating a higher risk of developing DM in alcohol-related pancreatitis in comparison with idiopathic pancreatitis [24]. But, several studies since have found no difference in risk of PPDM between aetiologies of pancreatitis [25,26]. A meta-analysis including 8,970 patients did not find alcohol-related CP to be a risk factor for the development of DM [27]. Smoking has not convincingly been established as a risk factor for PPDM but may contribute to worsening beta-cell function and insulin resistance [28]. Taken together it is important to maintain a high index of suspicion for PPDM in all patients with pancreatitis regardless of the aetiology or severity of the initial episode.

#### Recurrent pancreatitis

Even though the severity and aetiology of pancreatitis are not associated with PPDM, recurrent episodes of pancreatitis seem to pose a higher risk for PPDM. Specifically, two or more recurrences double the risk for PPDM with increasing risk with more episodes [8,29].

#### Disconnected pancreatic duct (DPD)

DPD can result in isolation of pancreatic parenchyma distal to duct disruption leading to atrophy and development of EPI and PPDM. Observational studies have reported a DM incidence ranging from 16%–52% in DPD [30]. Recent data shows that proximal disconnection has two times higher association with PPDM suggesting a correlation between pancreatic atrophy and DM development [31]. Close follow-up is imperative and early islet cell auto-transplantation has been suggested but further studies are needed [32].

In summary, patients with underlying risk factors (age <40 years, male gender, lean or overweight) who develop pancreatitis and consequent EPI appear to be at the highest risk for developing PPDM. The severity of the initial episode or a specific aetiology do not appear to be related to the development of PPDM. Clinical vigilance and regular follow up these patients are thus essential for prompt diagnosis.

## Diagnosis

At present, there are no consensus criteria for the diagnosis of PPDM. Ewald and Bretzel proposed a composite criterion for PPDM diagnosis consisting of diagnosis of DM with biochemical evidence of EPI, abnormal pancreatic imaging, and absence of type I DM-associated autoimmune markers [33]. However, these criteria have poor sensitivity as not all cases of PPDM have radiographic evidence of pancreatic injury or EPI [34]. The American Diabetes Association (ADA) criteria for DM diagnosis should be used to diagnose PPDM defined as haemoglobin A1C (HbA1C) ≥6.5% or fasting plasma glucose (FPG) >126 mg/dL (confirmed by repeat testing) after 90-days of an episode of AP without any known h/o diabetes or impaired fasting glucose. If HbA1c and FPG are known and were less than 6.5% and 126 respectively and increase to >6.5% and >126 mg/dL after 90-days of AP episode it is known as new-onset diabetes after pancreatitis (NODAP). A ninety-day window is given after an AP attack because early in the course of AP, FPG may be unreliable due to acute physiological stress, fluid resuscitation, and complications of AP. The glucose tolerance test (GTT), although reliable, is cumbersome and rarely performed these days.

Importantly, evidence of exocrine pancreatic dysfunction or radiological evidence of pancreatic damage is not necessary for the diagnosis of PPDM. This is important as even mild AP with minimal to no radiographic changes has a similar risk of developing PPDM as compared to those with severe AP [34]. Patients suspected to have NODAP should also be tested for T1DM autoantibodies as the same may have been precipitated by an attack of AP.

Another important aspect when diagnosing a pancreatitis patient with new-onset diabetes is a high suspicion of occult malignancy. Chronic pancreatitis is a risk factor for both diabetes and pancreatic cancer, and new-onset diabetes can be an earlier marker of pancreatic cancer [5,35,36]. Thin subjects who are >50 years old at the time of diabetes diagnosis, with sudden weight loss and severe hyperglycaemia are at the highest risk of developing pancreatic cancer, and clinicians should have a low threshold for abdominal imagining (CT or MRI/MRCP) in these patients [5].

## Management

Pancreatic inflammation leads to structural and functional loss of islet cell mass leading to loss of insulin, glucagon, and pancreatic polypeptide, which leads to the development of difficult to control diabetes with large fluctuations in blood glucose, which are difficult to control. In addition to the loss of glucagon response to hypoglycaemia, poor eating patterns due to pain and nausea, carbohydrate malabsorption, and alcohol use can also lead to labile blood glucose levels. A significant proportion of these patients also have concomitant EPI, leading to fat malabsorption with loss of fat-soluble vitamins. Loss of vitamin D can lead to metabolic bone disease and osteoporosis. The lack of luminal enzymes may also decrease the incretin response, further impairing glycemic control [37]. The management of PPDM is challenging and could be exhaustive for general practitioners taking care of these patients. Besides routine diabetic care including monitoring for nephropathy, retinopathy, and neuropathy they have to take care of pancreatitis-related complications including post-pancreatitis pain, poor oral intake, psychological issues, EPI, and vitamin and mineral deficiencies.

Managements of PPDM comprise of

1. Behavioural/Lifestyle interventions
  - Alcohol/substance abstinence
  - Nutrition
  - Exercise
  - Neuro/cognitive and psychiatric health
  - Pain control
  - Complementary and alternate therapies
2. Pharmacological Therapies:
  - Fiber
  - Pancreatic enzyme supplementation
  - Insulin and oral hypoglycaemic therapies
  - Osteoporosis treatment

### Behavioural/lifestyle modifications

- ***Alcohol Abstinence***: Alcohol can impair hepatic glucose production and can lead to hypoglycaemia, especially in diabetics on insulin therapy. Alcohol abstinence in patients with alcoholic pancreatitis can prevent EPI and reduce pain. Alcohol also tends to be associated with smoking and use of other recreational drug use which could either be a direct or indirect risk factor for pancreatitis and worsen its complications, along with pain, poor dietary habits, and medication non-compliance [38]. Beside routine counselling for alcohol cessation, patients at high risk of relapse to alcohol should be encouraged to join alcoholic anonymous (AA) groups [39]. Abstinence after an episode of acute alcoholic pancreatitis protects against recurrent attacks and pancreatic dysfunction is also rare among abstinent patients [40]. Patients at risk for hospital admission due to alcohol misuse may benefit from targeted interventions to increase rates of outpatient follow-up after hospital discharge [41].
- ***Nutrition***: Pancreatitis is a state of stress and catabolism and the energy expenditure could be up to 1.2–1.5 times than baseline. Any lag in this can lead to a state of malnutrition and decreased immunity leading to a poor intestinal mucosal barrier, increase in intestinal bacterial translocation, poor inflammatory response, and worse prognosis [42]. Patients with both acute and chronic pancreatitis are at risk of malnutrition. In a meta-analysis of 22 studies including 2,024 patients with AP, oral intake intolerance was noted in 16.3% [42]. Assessing for sarcopenia is another way of estimating malnutrition by assessing muscle strength, quality/quantity, and physical performance. Sarcopenia prevalence in CP ranges from 17%–64% and negatively affects the quality of life (QOL), increases hospitalizations and mortality. Sarcopenia and albumin levels have also been linked with EPI in patients with CP [43,44]. Another confounding factor is chronic nausea and pain in patients with pancreatitis which could lead to poor oral intake. Therefore, in patients with pancreatitis, it is important to assess and manage malnutrition aggressively. In this review, we will focus on dietary therapies for PPDM.

Currently, ADA does not recommend a specific diet for the management of PPDM. The strongest evidence exists for the Mediterranean, low-fat, or carbohydrate diets with a recommendation to use whole foods rather than processed items. The dietary pattern should be developed by taking into consideration individual geographic, cultural, and financial factors to ensure compliance [45]. Macronutrient distribution should be individualized based on preferences and metabolic goals [46]. The American Gastroenterology Association recommends a low-fat diet (less than 30% of total calories from fat) to reduce steatorrhoea and pain in patients with pancreatitis [47]. In addition, there are some data about the beneficial effect of a low-fat elemental diet to decrease pain in patients with CP [48].

However, in patients with PPDM, there are some challenges with a low-fat diet. Decreased fat intake can lead to an increase in carbohydrate intake potentially worsening hyperglycaemia. Furthermore, excessive protein to compensate for a low-fat diet can cause ketosis, and long-term adherence and compliance to a low-fat diet are yet to be established. Therefore, despite dietary recommendations for patients with pancreatitis, it is difficult to maintain the proper dietary balance to achieve glycemic and pain control in PPDM. All patients with PPDM should be evaluated and followed by an expert dietician to assess for malnutrition and discuss low-fat diet options. We suggest an individualized dietary plan based on glycemic control, fluctuations in blood glucose, dietary tolerance, and the overall health of the patient. Physicians should evaluate for EPI and micronutrient deficiencies and replete appropriately.

- ***Exercise***: Besides improving sarcopenia, regular exercise regulates insulin sensitivity and overall systemic metabolism by activating metabolic changes in the liver, adipose tissue, vasculature, and pancreas [49,50]. Regular exercise improves insulin resistance, a critical factor in pathogenesis of PPDM and improves beta-cell function defined as the “disposition index” [51,52]. Muscle released interleukin-6 (IL-6) and the peroxisome proliferator-activated receptor γ coactivator lα (PGClα)-dependent myokine irisin have been shown to have a protective effect against proinflammatory-induced beta-cell loss and beta-cell apoptosis induced by lipotoxic conditions [53]. Although, well established in T2DM, exercise therapy remains to be evaluated in PPDM. Considering the significant benefits of regular exercise, PPDM patients should be encouraged to engage in moderate to vigorous aerobic activity as individually tolerated [54]. Future studies should investigate the role of exercise in improving glycemic control, malnutrition and pain in PPDM.
- ***Neurocognitive and psychiatric health***: Chronic pain associated with anxiety and depression are prevalent amongst patients with pancreatitis [55]. In a study of 171 patients with CP, anxiety was present in 46.8% and depression in 38.6%, with an overlap in 29%. These patients also had higher pain prevalence and severity, reduced global health scores, and functional subscales (*p*<0.01). This indirectly suggests a higher likelihood of these disorders in patients with PPDM [56]. In addition, diabetic people are at increased risk of cognitive decline, since the metabolic and vascular disturbances of the disease affect brain function [57]. Depression or diabetes-related distress are common in diabetes and the majority of them remain undiagnosed and untreated. In a meta-analysis of 248 studies (>83 million participants), depression was observed in 28% of patients with diabetes. Screening for mood disorders should be undertaken early in PPDM as it has been shown that early diagnosis can improve healthcare-related quality of life in diabetes [58]. Studies have confirmed that treatment focussing on pain and diabetes may alleviate depressive symptoms [59].
- ***Pain***: As mentioned above, in patients with PPDM, the presence of chronic pain can lead to substance abuse, poor dietary and medical compliance, and psychiatric issues. In addition to alcohol and smoking cessation, a multidisciplinary pain management strategy with prompt referral to a chronic pain specialist is imperative. Pain management should focus on reducing non-steroidal anti-inflammatory drug and opioid use, ruling out other aetiologies of pain (e.g. small intestinal bacterial overgrowth, cannabinoid/cyclic vomiting syndrome, etc.), and encouraging utilization of complementary therapies including yoga and acupuncture [60]. Dietary compliance with a low-fat diet and replacement with pancreatic enzyme supplementation has also been shown to reduce pain [61]. Endoscopic interventions (celiac plexus block, pancreatic duct sphincterotomy, or shock wave lithotripsy) should be reserved for patients with persistent pain despite the above measures or with pancreatic duct strictures and stones [60].
- ***Complementary and alternative therapies****:* Yoga, acupuncture, and Ayurveda have been reported to improve glycemic control and chronic pain [62,63,64], and thus theoretically may provide dual benefit in PPDM. A meta-analysis of nine randomized controlled trials showed yoga decreased FPG by up to 28 mg/dl in 411 T2DM patients. Yoga in 30 patients with pancreatitis has been shown to decrease alcohol dependence, stress, and overall quality of life [65]. This may be beneficial in patients with PPDM by increasing healthy habits and potentially better DM control. However, studies in patients with pancreatitis or PPDM are lacking. Acupuncture in the management of pain related to pancreatitis is an evolving field and recent data in 15 patients with CP demonstrated pain relief compared with placebo but the effect was short-lived with similar pain scores 1 week after treatment [63]. Currently these may be used as an adjunct to conventional therapies in the management of PPDM.

### Pharmacological therapies

- ***Dietary Fiber*:** Dietary fiber intake is known to improve blood sugar control and decrease HbA1c levels in patients with T2DM and decrease overall mortality [66]. Recently a study of 36 patients with PPDM showed an association between dietary fiber intake and reduced FPG [66]. Fiber intake was determined by the EPIC-Norfolk food questionnaire that assessed dietary patterns over a year before the study visit. The mean time for study visit from the last attack of AP was 27.0 months. The inverse association between fiber intake and FPG persisted after adjustment for several factors including demographic and pancreatitis-specific features such as aetiology, presence of necrosis, and recurrence of pancreatitis. Randomized studies should evaluate the role of dietary fiber in PPDM to derive a strong recommendation.
- ***Pancreatic enzyme supplementation*:** The major goals of medical or nutrition therapy in patients with PPDM are to prevent malnutrition, control symptoms of steatorrhoea, and minimize meal-induced hyperglycaemia. In PPDM patients with EPI, supplementation with pancreatic enzymes can decrease steatorrhoea by improved fat absorption. EPI has been reported in up to 50% of diabetics with higher prevalence in patients with more severe DM [67]. Pancreatic enzyme supplementation prevents loss of fat-soluble vitamins A, D, E, and K. Secretion of intestinal-derived incretin is also improved by pancreatic enzymes supplementation which is associated with improved insulin secretion and better glucose control during meals [38]. In a retrospective, post hoc, subgroup (±DM) analysis of a double-blind, randomized, placebo-controlled trial of pancrelipase in patients with EPI, pancrelipase improved fat and protein absorption in patients with EPI and diabetes [68]. In addition to ruling out other causes of steatorrhoea, it is imperative to avoid the common issues with pancreatic enzymes supplementation including inappropriate dosing and timing, not taking with snacks, unequal calorie distribution during meals, using uncoated preparations, and not taking with acid-suppressive medications [69,70]. These issues can be mitigated by collaborative counselling efforts by primary care, gastroenterologist, endocrinologist, and dietician. A routine-close follow is also necessary to monitor and adjust dosing based on eating patterns and blood glucose levels.
- ***Oral hypoglycemics and insulin therapy*:** Although there has been significant development in the management of T2DM over the last two decades, no specific guidelines exist currently for the management of PPDM. Moreover, most major trials for pharmacotherapy for T2DM exclude patients with pancreatitis limiting the applicability of these drugs to this special population. There are no randomized control trials that have compared the efficacy and safety of available anti-diabetic medications in PPDM.

Current evidence for the management of PPDM is extrapolated from retrospective and population-based observational studies [71]. Blood glucose is difficult to control in PPDM and early referral to endocrinology for medication management may be beneficial.

As insulin deficiency is a critical feature of PPDM, insulin therapy is utilized in most patients. Disease severity and duration are directly related to insulin deficiency [38]. A recent study from the United Kingdom showed that compared to patients with T2DM, patients with PPDM are 10-times (13.4% vs 1.4%) more likely to require insulin at one-year post DM diagnosis [4]. Another study from Denmark, showed that patients with PPDM were 4-times more likely to be prescribed insulin in comparison with patients with T2DM [72]. Although utilized more frequently, insulin therapy has not yielded better glycemic control or provided mortality benefit in PPDM in retrospective observational studies [4,73].

Selection of oral anti-diabetic therapy is nuanced and data are lacking in the PPDM population. The COSMOS group evaluated management strategies in a nationwide study from New Zealand [73]. Using the pharmaceutical dispensing data over 10 years (2006–2015), 836 patients with PPDM were included. Metformin use was associated with significantly lower mortality risk (OR: 0.51, *p*<0.05) in comparison with no anti-diabetic treatment. The mortality benefit persisted on sensitivity analysis excluding patients with <6 months of follow-up to reduce the risk of reverse causation. A dose-response analysis suggested maximum benefit at a dose of 1,000 mg/day. A postulated benefit of metformin in this population is a reduced risk of pancreatic adenocarcinoma in a susceptible population which might have contributed to the lower mortality [74,75]. Despite these benefits, the major side effects of metformin including nausea, abdominal discomfort, diarrhoea, and weight loss make the use of Metformin challenging in this patient population [76]. Similarly, the use of thiazolidinediones to improve insulin sensitivity is limited by its side effects, particularly congestive heart failure and increased risk of osteoporosis [77].

At present there is insufficient evidence to either recommend for or against newer therapies and decisions should be individualized. Newer therapies including glucagon-like peptide (GLP-1) analogues and sodium glucose cotransporter-2 inhibitors (SGLT-2) have revolutionized the treatment of T2DM by achieving weight loss, improving insulin sensitivity, and improving cardiovascular and renal outcomes [78]. However a reported side effect of GLP-1 analogues is pancreatitis itself and a postulated higher risk of pancreatic cancer [79]. Although this risk has not found to be higher in T2DM in recent meta-analyses, the studies did not include patients with a prior history of pancreatitis [80,81]. Another challenge is the associated weight loss with GLP-1 analogues which can theoretically worsen malnutrition in PPDM patients [82,83]. It is important to note that even with these concerns, a population-based study from Denmark found that 20% of patients with PPDM were prescribed incretin-based therapies [72]. There are currently no studies that have evaluated the clinical course of patients specifically with GLP-1 analogue related AP and the risk of PPDM. In addition, no studies have evaluated the use of this class of drugs in patients with PPDM and the risk of future episodes of AP. Similarly, data for SGLT-2 inhibitors is lacking as studies have excluded patients with PPDM. There is an increased risk of diabetic ketoacidosis in states of insulin deficiency with SGLT-2 inhibitor drug class. Until studies are conducted in patients with PPDM, a recommendation for or against use of agents in this drug class cannot be made.

- ***Osteoporosis*:** All patients with PPDM should be screened with DXA for osteoporosis as both AP and CP are risk factors for vitamin D deficiency, osteoporosis, and fractures [84,85]. In addition to identifying and treating modifiable risk factors (alcohol, tobacco, low BMI, EPI, and chronic opioid use), Vitamin D and calcium supplementation should be started to treat and prevent osteopenia, osteoporosis, and lower the risk of fracture [86]. Patients with established bone disease should be thoroughly evaluated for appropriate therapies by an endocrinologist [85,86].

**Figure 1.**
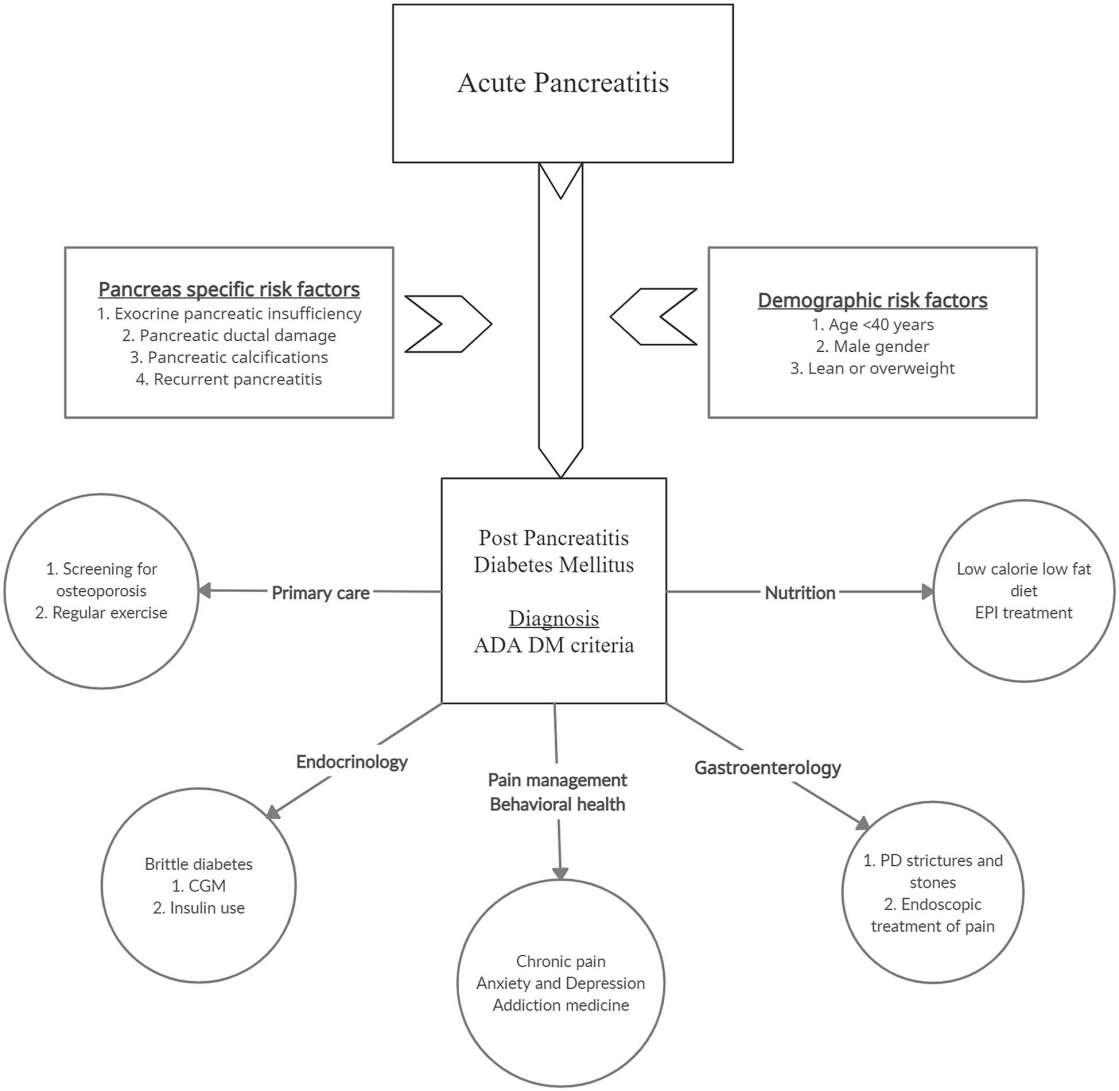
Multidisciplinary care model for patients with post-pancreatitis diabetes mellitus. Abbreviations: ADA: American Diabetes Association; CGM: Continuous glucose monitoring; DM; Diabetes mellitus; EPI: Exocrine pancreatic insufficiency; PD: Pancreatic duct.

In conclusion, PPDM, an increasingly recognized complication of pancreatitis is now the second most common cause of new-onset DM. Current evidence suggests patients with pancreatitis, regardless of aetiology or severity, can develop PPDM. Patients with underlying risk factors (age <40 years, male gender, lean or overweight) who develop pancreatitis and consequent EPI appear to be at the highest risk for developing PPDM. New-onset diabetes in patients with pancreatitis could also be an early marker of occult pancreatic malignancy. Management of PPDM is complex and requires a team-based approach including gastroenterologists, endocrinologists, primary care physicians, nutritionists, and behavioural health specialists (Figure 1). In addition to micro-and macrovascular complications of DM, identification, and treatment of EPI, malnutrition, and chronic pain, concomitant psychological ailments are imperative for holistic care. Early referral to endocrinology is critical to managing difficult to control diabetes with an evolving role of continuous glucose monitoring (CGM). Future research to study the effect of pharmacological therapy in PPDM is required as our understanding of pathophysiology improves. Prospective studies currently underway will help us understand the risk of both pancreas and DM-specific complications in these patients.

## Data Availability

Data sharing does not apply to this article as no new data were created or analysed in this study.

## Abbreviations

ADA: American Diabetes Association
AP: Acute pancreatitis
aOR: Adjusted odds ratio
BMI: Body mass index
CP: Chronic pancreatitis
CFRD: Cystic fibrosis related diabetes
DEP: Diabetes of exocrine pancreas
DM: Diabetes mellitus
DPD: Disconnected pancreatic duct
EPI: Exocrine pancreatic insufficiency
FPG: Fasting plasma glucose
GLP-1: Glucagon-like peptide-1
GRS: Genetic risk scores
GTT: Glucose tolerance test
HbA1C: Haemoglobin A1C
; HR: Hazard ratio
IL-6: Interleukin-6
NODAP: New-onset diabetes after pancreatitis
T1DM: Type 1 diabetes mellitus
T2DM: Type 2 diabetes mellitus
PPDM: Post pancreatitis diabetes mellitus
PGClα: Peroxisome proliferator-activated receptor γ coactivator lα
QOL: Quality of life
